# Impact of Preoperative Nutritional Assessment on Other-Cause Survival after Gastrectomy in Patients with Gastric Cancer

**DOI:** 10.3390/nu15143182

**Published:** 2023-07-18

**Authors:** Ryota Matsui, Noriyuki Inaki, Toshikatsu Tsuji

**Affiliations:** 1 Department of Gastroenterological Surgery, Ishikawa Prefectural Central Hospital, 2-1 Kuratsuki-higashi, Kanazawa 920-8530, Ishikawa, Japan; n.inaki@viola.ocn.ne.jp (N.I.); toshi_toshi25@yahoo.co.jp (T.T.); 2 Department of Gastrointestinal Surgery/Breast Surgery, Graduate School of Medical Science, Kanazawa University, 13-1 Takara-machi, Kanazawa 920-8641, Ishikawa, Japan

**Keywords:** body composition, gastric cancer, malnutrition, other-cause survival, prognosis

## Abstract

This study aimed to clarify the factors associated with death due to other diseases after a gastrectomy for gastric cancer. This retrospective cohort study included consecutive patients who had undergone gastrectomy between April 2008 and June 2018 for primary stage II–III gastric cancer. The primary outcome was other-cause survival. To identify prognostic factors for other-cause survival for univariate analysis, we used a Cox proportional hazard regression model. A total of 512 patients met the inclusion criteria. The average age was 67.93 years, and the average body mass index was 22.75 kg/m^2^, with 84 (16.4%) being moderately malnourished and 88 (17.2%) being severely malnourished, as defined by the Global Leadership Initiative on Malnutrition (GLIM) criteria. The other-cause survival for the malnourished group was significantly worse than that for the normal group (*p* < 0.001). The prognosis was worse when the severity of malnutrition was worse (*p* < 0.001). Multivariate analysis showed that severe malnutrition was significantly independent of prognostic factors for other-cause survival (hazard ratio: 3.310; 95% confidence interval: 1.426–7.682; *p* = 0.005). Undernutrition, as defined by the GLIM criteria, is useful for the preoperative prediction of death due to other diseases after gastrectomy in patients with advanced gastric cancer.

## 1. Introduction

Recently, body composition, including skeletal muscle mass and fat mass, has been used to assess undernutrition, and its correlation with the prognosis for patients with gastric cancer after a gastrectomy has been explored [1,2,3]. The widely accepted Global Leadership Initiative on Malnutrition (GLIM) criteria consider reduced muscle mass to be indicative of undernutrition, suggesting that patients with sarcopenia are undernourished [4]. A recent systematic review has shown that reduced skeletal muscle mass is associated with a poor prognosis for gastric cancer patients [1,2,5], and reduced visceral and subcutaneous fat mass is also associated with a poor prognosis for gastric cancer patients [3,6,7]. Therefore, the assessment of body composition is essential for predicting postoperative outcomes.

Death due to other diseases is one factor related to long-term prognosis after gastrectomy; however, few studies have examined the relationship between death due to other diseases and undernutrition. Sarcopenia has been reported to increase short-term postoperative pneumonia [5]. In addition, the preoperative assessment of muscle quantity and quality, in particular, has been reported to be useful in assessing long-term death due to other diseases [8]. However, the effect of visceral and subcutaneous fat on death due to other diseases has not been clarified, and the relationship between undernutrition and death due to other diseases, as defined by the GLIM criteria, has not been fully investigated. The preoperative prediction of death due to other diseases after a gastrectomy is an important factor in postoperative treatment and its selection.

This study aimed to clarify the factors associated with death due to other diseases after a gastrectomy for advanced gastric cancer. In addition to cancer-related and surgical factors, we focused on body composition and undernutrition as defined by the GLIM criteria, which have recently drawn attention.

## 2. Materials and Methods

### 2.1. Study Design

This retrospective cohort study included consecutive patients who had undergone a gastrectomy between April 2008 and June 2018 for primary stage II–III gastric cancer, as defined by the 15th edition of the Japanese Classification of Gastric Carcinomas [9]. Patients were excluded if they (1) had residual gastric cancer, (2) had cancer in other organs, (3) had undergone surgical procedures not related to gastrectomy, (4) had unresectable distant metastases, and (5) had undergone preoperative treatment. Patients with positive ascite cytology (CY1) without distant metastases were included.

In this study, all experimental protocols were approved by the Ishikawa Prefectural Central Hospital Institutional Ethical Review Committee (authorization number 1847); they met the ethical guidelines issued by the Ministry of Health, Labour and Welfare for Medical and Health Research Involving Human Subjects; and they adhered to the Declaration of Helsinki. To provide all patients with the opportunity to decline participation, an opt out recruitment method was used.

### 2.2. Outcomes and Analyses

Other-cause survival (OCS), defined as the time between surgery and death not related to the gastric cancer, was the primary outcome of this study. We included deaths of patients without a recurrence of gastric cancer after a gastrectomy in the OCS. Kaplan–Meier survival analysis was performed using the log-rank test for OCS. To identify prognostic factors for OCS for univariate analysis, we used a Cox proportional hazard regression model, in which multivariate analysis was conducted to obtain hazard ratios (HRs). Statistical analyses were performed using EZR software (ver. 1.61) [10] and statistical significance was set at *p* < 0.05.

### 2.3. Definition of Other Factors

The GLIM criteria were used in this study to define the parameters used to diagnose the severity of malnutrition [4]. The body mass index (BMI) and body weight loss (BWL) rate were used to classify the patients as having moderate or severe malnutrition, according to the GLIM criteria (Table 1). Normal nutrition was defined as the absence of malnutrition.

Visual analysis of preoperative plain computed tomography (CT) images using the graphical analysis software Ziostation (ZIOSOFT, Tokyo, Japan) was used to estimate visceral and subcutaneous fat mass at the umbilical level, as well as skeletal muscle mass at the third lumbar vertebra level. Visceral and subcutaneous fat mass and skeletal muscle mass were measured on one CT image slice. The masses were divided by the height of the patient to determine the visceral adipose tissue index (VAI), subcutaneous adipose tissue index (SAI), and skeletal muscle mass index (SMI), respectively [11]. As performed in previous studies, we measured the CT values (in Hounsfield units (HU)) of the regions of interest at the umbilical level and then calculated the intramuscular adipose tissue content (IMAC) by dividing the CT value of the multifidus muscles by the CT value of the subcutaneous fat [12]. The cutoff values for the VAI, SAI, and IMAC were estimated for men and women based on the median values for each group, and the reported cutoff values for SMI were adopted [13]. The cutoff values for each parameter are presented in Table 2. Patients with an SMI below or above the cutoff value were classified as having a low SMI or a high SMI, respectively. Low SMI was further divided into moderate and severe SMI [13].

We defined postoperative complications that occurred within 30 days after surgery as Clavien–Dindo classification (CD) grade ≥ 2 and severe complications as CD grade ≥ 3 [14].

For comorbidities, chronic kidney disease was defined as an estimated glomerular filtration rate of < 60 mL/min/1.73 m^2^, diabetes was defined as either having a history of treatment or preoperative HbA1c of ≥ 6.5%, chronic obstructive pulmonary disease (COPD) was defined as an FEV1.0% of < 70% on spirometry, and congestive heart failure was defined as either having a history of treatment or ejection fraction of < 50% on echocardiography.

### 2.4. Postoperative Chemotherapy with S-1

Following the Japanese gastric cancer treatment guidelines [15], we administered postoperative adjuvant chemotherapy with S-1 to patients with cancer stages II or higher. The regimen was started at 80–120 mg/day and administered for 4 weeks, followed by 2 weeks of rest. If side effects were observed, we reduced the dose gradually according to the guidelines from 120 to 100 mg/day or from 100 to 80 mg/day. We decided to discontinue treatment when side effects could not be controlled with dose optimization, when there were two or more steps of dose reduction, or when there was a confirmed recurrence of disease during adjuvant chemotherapy. In this study, we defined treatment failure discontinuation of adjuvant chemotherapy within one year of having started it.

### 2.5. Postoperative Follow-Up

The postoperative follow-up was conducted at an outpatient clinic. Hematological tests were per formed at least every 2–3 weeks during the S-1 treatment, and at least every 3 months for 5 years after completion of the S-1 treatment. Patients underwent a CT scan every 6 months, and endoscopy every year, for 5 years after surgery. We administered no treatment other than adjuvant chemotherapy with S-1 until recurrence.

### 2.6. Clinicopathological Variables

The prognostic factors that were analyzed were sex, age, BMI, surgical approach, surgical procedure, lymph node dissection, pathological stage, postoperative chemotherapy, comorbidities, GLIM malnutrition, SMI, IMAC, VAI, SAI, and postoperative complications. We performed a multivariate analysis of these factors, including those with *p* < 0.05 in univariate analyses to identify prognostic factors.

## 3. Results

### 3.1. Patient Background

Table 3 presents a summary of patient characteristics. A total of 512 patients (336 (65.6%) male and 176 (34.4%) female) met the eligibility criteria. The average age was 67.93 years old and the average BMI was 22.75 kg/m^2^. The pathological stages of the patients were as follows. Briefly, 88 (17.2%) were stage I, 176 (34.4%) were stage II, 193 (37.2%) were stage III, and 55 (10.7%) were stage IV. Eighty-four (16.4%) patients were moderately malnourished and eighty eight (17.2%) were severely malnourished. The low-SMI group comprised 235 (48.5%) patients, while the moderate and severe groups comprised 152 (31.3%) and 83 (17.1%), respectively. There were 246 (50.7%) in the high-IMAC group, 243 (50.1%) in the low-VAI group, and 242 (49.9%) in the low-SAI group.

### 3.2. Comparison of OCS Curves

**Figure 1:**
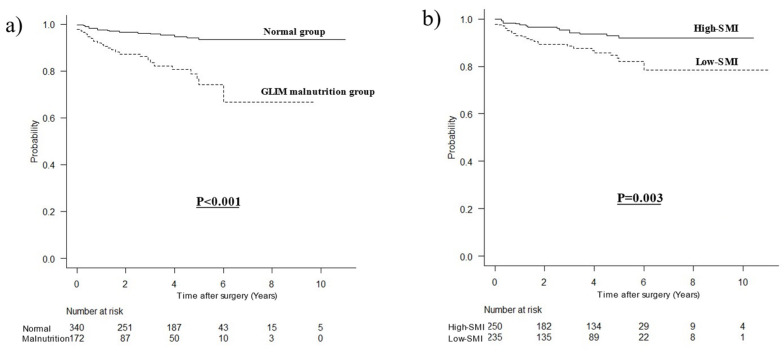

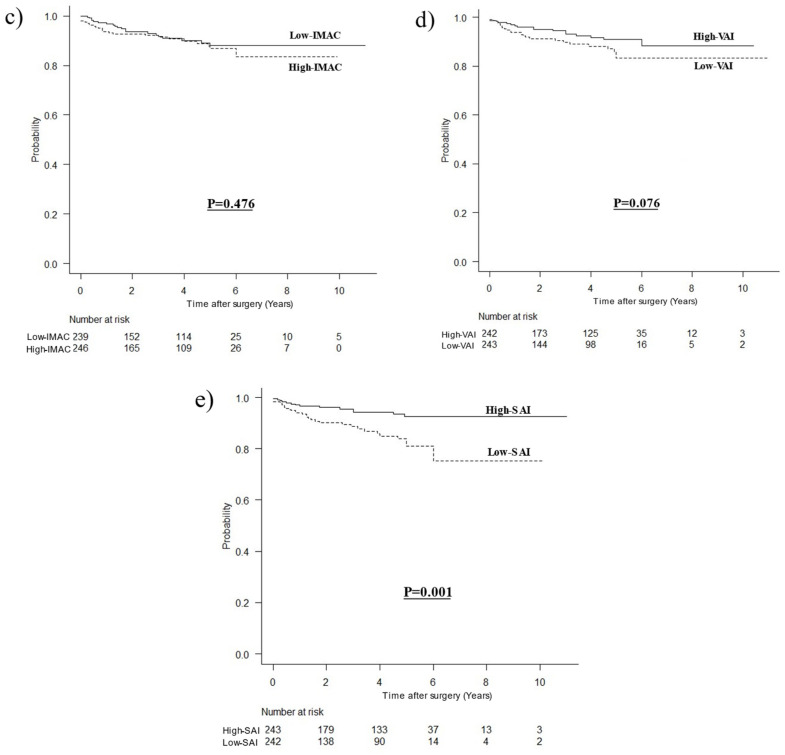
Other-cause survival curves with respect to the following factors: (**a**) malnutrition defined by GLIM criteria (*p* < 0.001), (**b**) SMI (*p* = 0.003), (**c**) IMAC (*p* = 0.476), (**d**) VAI (*p* = 0.076), (**e**) and SAI (*p* = 0.001).

Death due to other diseases was observed in 45 (8.8%) patients. The median follow-up time was 41 months (interquartile range: 17–60 months). The OCS curves of the two groups are compared in Figure 1. The prognosis for the malnourished group was significantly worse than that for the normal group (*p* < 0.001), that for the low-SMI group was significantly worse than that for the high-SMI group (*p* = 0.003), and that for the low-SAI group was significantly worse than that for the high-SAI group (*p* = 0.001). In contrast, the prognoses for the high- and low-IMAC groups (*p* = 0.476) and the low- and high-VAI groups were not significantly different (*p* = 0.076).

### 3.3. Stratified Survival Curves for OCS

**Figure 2:**
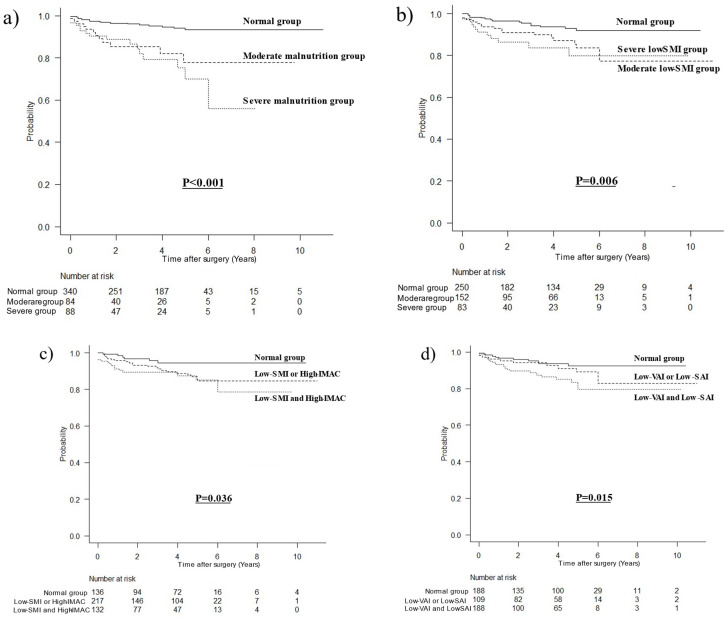
Other-cause survival curves stratified with respect to the following factors: (**a**) severity of malnutrition defined by GLIM criteria (*p* < 0.001), (**b**) low-SMI severity (*p* = 0.006), (**c**) low SMI and/or high IMAC (*p* = 0.036), and (**d**) low VAI and/or low SAI (*p* = 0.015).

The stratified survival curves for OCS are shown in Figure 2. As can be seen from Figure 2a, the prognosis worsened with the increasing severity of malnutrition (*p* < 0.001). Figure 2b shows that both moderate and severe low-SMI patients had a poor prognosis (*p* = 0.006). Figure 2c shows that patients with the comorbidities of low SMI and high IMAC had the worst prognosis (*p* = 0.036). Finally, Figure 2d shows that the patients with both low VAI and low SAI had the worst prognosis (*p* = 0.015).

### 3.4. Prognostic Factors for OCS

Table 4 presents the results of the analysis of the prognostic factors for OCS. Univariate analysis showed that an age of ≥ 70 years (*p* < 0.001), D2 lymph node dissection (*p* = 0.038), postoperative chemotherapy (*p* < 0.001), moderate and severe malnutrition (*p* = 0.013 and *p* < 0.001, respectively), severely low SMI (*p* = 0.024), low SAI (*p* = 0.002), and severe postoperative complications (*p* < 0.001) were statistically significant prognostic factors for OCS. Multivariate analysis showed that postoperative chemotherapy (HR: 0.283; 95% confidence interval (CI): 0.140–0.573; *p <* 0.001), severe malnutrition (HR: 3.310; 95% CI: 1.426–7.682; *p* = 0.005), and severe postoperative complications (HR: 3.353; 95% CI: 1.707–6.588; *p* < 0.001) were significant independent prognostic factors for OCS.

## 4. Discussion

Our study identified factors associated with death due to other diseases after a gastrectomy in patients with advanced gastric cancer. We found that severe malnutrition, as defined by the GLIM criteria and assessed via the BMI and BWL, as a preoperative predictor and the occurrence of severe complications with CD grade 3 or higher as a postoperative factor were independent factors associated with a poor prognosis for OCS.

In this study, pneumonia was the most common cause of death among the other diseases. GLIM-defined malnutrition has been linked to death due to other diseases caused by pneumonia, which is a complication of gastric cancer and the risk of which increases as undernutrition increases [13]. In addition, severe GLIM-defined undernutrition not only increases postoperative pneumonia but also increases mortality within 90 days after surgery [16]. The present study showed that severe GLIM-defined undernutrition increases the incidence of fatal pneumonia in the long term. This is the first study to show that GLIM-defined undernutrition is a factor associated with a poor prognosis for OCS.

Analysis of body composition revealed that muscle quantity, muscle quality, visceral fat mass, and subcutaneous fat mass were not independent indicators of OCS. Comparison of the survival curves showed that the comorbidities of low SMI and high IMAC were factors for a poor prognosis, as were low VAI and low SAI. A previous report showed the usefulness of the assessment of muscle quantity and quality in predicting death due to other diseases [8], supporting the results of this study. We used the SMI cutoff value, which is the most commonly used value in Asia, and further divided the low SMI category into moderate and severe SMI, but none was an independent predictor of a poor prognosis. Based on these results, the combination of muscle mass and muscle quality is more useful than muscle mass and muscle quality separately for predicting the prognosis for OCS. The assessment of handgrip strength has been regarded as essential for the diagnosis of sarcopenia [17]. In an earlier study, we showed that low preoperative handgrip strength increases the risk of death due to other diseases [18]. In addition to muscle mass and muscle quality measurements, functional assessments may be useful in predicting death due to other diseases, but further study is needed. Although fat mass reflects excessive nutrient accumulation, BWL occurs after gastrectomy. Those with a low VAI and low SAI before gastrectomy may experience postoperative energy depletion. This suggests that body composition assessment alone cannot predict death due to other diseases.

Regarding the generalization of our results, the determination of GLIM-defined malnutrition based on the BMI and BWL does not require any special tests and can be easily performed immediately in daily clinical practice. In addition, GLIM-defined malnutrition can be evaluated repeatedly, not only preoperatively, but also during the follow-up period. In this study, we did not include low SMI in the diagnosis of GLIM-defined malnutrition because the cutoff value for muscle mass has not been established in the current GLIM criteria, so its validity has not been verified [19]. Multivariate analysis suggested that GLIM-defined malnutrition based on BMI and BWL may be more useful than muscle mass alone in predicting death due to other diseases.

Severe complications were a poor prognostic factor related to OCS. Previous reports have shown that postoperative complications worsen long-term prognosis [20,21]. In addition, Nagata et al. show that the occurrence of severe complications increases death from other diseases [22]. The results of this study also support these findings.

This study has some limitations. First, it was a single-center retrospective cohort study. Second, the cutoff values of the parameters are unclear and require validation via additional multicenter cohort studies. This study revealed that a low preoperative BMI or high BWL may increase the risk of death due to other diseases in gastric cancer patients with postoperative weight loss. Patients with preoperative GLIM-defined malnutrition should be followed up to check their nutritional status and, if necessary, considered for nutritional support. They should also be followed up for any postoperative decline in physical function. Intervention with physical exercise may be necessary to prevent long-term pneumonia and muscle weakness due to lack of use. Therefore, it is necessary to clarify whether or not such nutritional and exercise interventions have prolonged prognostic effects on patients with GLIM-defined malnutrition.

## 5. Conclusions

Undernutrition as defined by the Global Leadership Initiative on Malnutrition criteria as useful for the preoperative prediction of death due to other diseases after gastrectomy in patients with advanced gastric cancer.

## Figures and Tables

**Table 1:** Malnutrition severity grade as defined by GLIM criteria in the present study.

	BWL (%)	BMI (kg/m^2^)	Skeletal Muscle Mass
Moderate malnutrition	5–10% within past 6 months prior to surgery or 10–20% beyond 6 months prior to surgery	<20.0 if <70 years old or <22.0 if ≥70 years old	Not available
Severe malnutrition	>10% within past 6 months prior to surgery or >20% beyond 6 months prior to surgery	<18.5 if <70 years old or <20.0 if ≥70 years old	Not available

*GLIM*: Global Leadership Initiative on Malnutrition; *BMI*: body mass index; *BWL*: body weight loss.

**Table 2:** Cutoff values of body composition parameters in present study.

Parameters		Cutoff Values		
		Male	Female	Prevalence (%)
Low SMI (cm^2^/m^2^)	Moderate	<40.8	<34.9	31.3%
	Severe	<34.5	<28.9	17.1%
High IMAC		>−0.42	>−0.32	50.7%
Low VAI (cm^2^/m^2^)		<35.42	<26.81	50.1%
Low SAI (cm^2^/m^2^)		<33.90	<41.70	49.9%

*IMAC*: intramuscular adipose tissue content; *SAI*: subcutaneous adipose tissue index; *SMI*: skeletal muscle mass index; *VAI*: visceral adipose tissue index.

**Table 3:** Patient characteristics.

	All Patients (*n* = 512)
Sex	
Male	336 (65.6%)
Female	176 (34.4%)
Age (mean ± SD)	67.93 ± 11.10
BMI (mean ± SD)	22.75 ± 3.52
Surgical approach	
Laparoscopic	266 (52.0%)
Open	246 (48.0%)
Surgical procedure	
Distal gastrectomy	279 (54.5%)
Proximal gastrectomy	25 (4.9%)
Total gastrectomy	208 (40.6%)
Lymph node dissection	
D1+	233 (45.5%)
D2	279 (54.5%)
Clinical stage	
II	163 (31.8%)
III	349 (68.2%)
Pathological stage	
I	88 (17.2%)
II	176 (34.4%)
III	193 (37.7%)
IV	55 (10.7%)
Postoperative chemotherapy	326 (63.7%)
Comorbidity	
CKD	93 (18.2%)
COPD	110 (21.5%)
Diabetes	92 (18.0%)
CHF	28 (5.5%)
Preoperative albumin (g/dL)	
>3.5	409 (82.6%)
≤3.5	86 (17.4%)
Preoperative CRP (mg/dL)	
<0.5	423 (82.6%)
≥0.5	89 (17.4%)
Malnutrition defined by GLIM criteria	
Moderate	84 (16.4%)
Severe	88 (17.2%)
SMI (cm^2^/m^2^), median (IQR)	39.08 (33.98–45.33)
Low SMI (all patients)	235 (48.5%)
Low SMI (moderate)	152 (31.3%)
Low SMI (severe)	83 (17.1%)
IMAC, median (IQR)	−0.39 (−0.47 to −0.28)
High IMAC	246 (50.7%)
VAI (cm^2^/m^2^), median (IQR)	32.46 (16.69–51.02)
Low VAI	243 (50.1%)
SAI (cm^2^/m^2^), median (IQR)	36.32 (21.70–53.83)
Low SAI	242 (49.9%)

*BMI*: body mass index; *CHF*: chronic heart failure; *CKD*: chronic kidney disease; *COPD*: chronic obstructive pulmonary disease; *CRP*: C-reactive protein. *GLIM*: Global Leadership Initiative on Malnutrition; *IMAC*: intramuscular adipose tissue content; *IQR*: interquartile range; *SD*: standard deviation; *SAI*: subcutaneous adipose tissue index; *SMI*: skeletal muscle mass index; *VAI*: visceral adipose tissue index.

**Table 4:** Results of analyses of prognostic factors for other-cause survival.

Variables	Univariate Analysis			Multivariate Analysis		
	HR	95% CI	*p* Value	HR	95% CI	*p* Value
Sex						
Female	1					
Male	1.186	0.631–2.230	0.597			
Age (years)						
<70	1			1		
≥70	3.669	1.918–7.020	<0.001	1.709	0.820–3.562	0.153
Surgical procedure Distal gastrectomy	1					
Total gastrectomy	0.802	0.431–1.493	0.487			
Surgical approach						
Laparoscopic	1					
Open	1.278	0.712–2.296	0.411			
Lymph node dissection						
D1+	1			1		
D2	0.534	0.295–0.965	0.038	0.736	0.392–1.382	0.34
*p* stage						
<III	1					
>III	0.854	0.465–1.567	0.61			
Postoperative chemotherapy Absent	1			1		
Present	0.204	0.107–0.388	<0.001	0.283	0.140–0.573	<0.001
CKD						
Absent	1					
Present	1.406	0.696–2.839	0.342			
Diabetes						
Absent	1					
Present	1.769	0.914–3.427	0.091			
COPD						
Absent	1					
Present	1.735	0.910–3.309	0.095			
CHF						
Absent	1					
Present	2.201	0.867–5.586	0.097			
GLIM malnutrition						
Normal	1			1		
Moderate	2.316	1.193–4.496	0.013	2.100	0.904–4.880	0.085
Severe	3.350	1.813–6.191	<0.001	3.310	1.426–7.682	0.005
SMI (cm^2^/m^2^)						
High	1			1		
Low (moderate)	1.559	0.858–2.831	0.145			
Low (severe)	2.149	1.107–4.173	0.024	1.121	0.547–2.297	0.756
IMAC						
Low	1					
High	1.238	0.688–2.229	0.477			
VAI (cm^2^/m^2^)						
High	1					
Low	1.706	0.938–3.102	0.08			
SAI (cm^2^/m^2^)						
High	1			1		
Low	2.698	1.431–5.086	0.002	1.876	0.897–3.925	0.095
Postoperative complications						
Absent	1			1		
Total	3.230	1.794–5.815	<0.001			
Severe	4.797	2.551–9.022	<0.001	3.353	1.707–6.588	<0.001

*BMI*: body mass index; *CHF*: chronic heart failure; *CI*: confidence interval; *CKD*: chronic kidney disease; *COPD*: chronic obstructive pulmonary disease; *CRP*: C-reactive protein; *GLIM*: Global Leadership Initiative on Malnutrition; *HR*: hazard ratio; *IMAC*: intramuscular adipose tissue content; *IQR*: interquartile range; *SD*: standard deviation; *SAI*: subcutaneous adipose tissue index; *SMI*: skeletal muscle mass index; *VAI*: visceral adipose tissue index.

## Data Availability

The datasets generated and/or analyzed during the current study are available upon reasonable request from the corresponding author.
